# Mothers adhering to a vegan diet: feeding practices of their young children and underlying determinants — a qualitative exploration

**DOI:** 10.1017/jns.2025.14

**Published:** 2025-03-21

**Authors:** Josine Pereboom, Deidre Meulenbroeks, Sanne M.P.L. Gerards, Simone J.P.M. Eussen, Hubertina C.J. Scheepers, Daisy M.A.E. Jonkers, Jessica S. Gubbels

**Affiliations:** 1 Department of Health Promotion, NUTRIM Institute of Nutrition and Translational Research in Metabolism, Maastricht University, Maastricht, the Netherlands; 2 Maastricht University Medical Centre, Department of Obstetrics & Gynecology, GROW, Research Institute for Oncology and Developmental Biology, Maastricht, the Netherlands; 3 Maastricht University, NUTRIM Institute of Nutrition and Translational Research in Metabolism, Department of Internal Medicine, Division Gastroenterology-Hepatology, Maastricht, the Netherlands; 4 Department of Epidemiology, Care and Public Health Research Institute CAPHRI, Maastricht University, Maastricht, the Netherlands; 5 Cardiovascular Research Institute Maastricht (CARIM), Maastricht University, Maastricht, the Netherlands

**Keywords:** Children, Feeding practices, Mothers, Plant-based diet, Vegan diet, VD, vegan diet (i.e., strict plant-based diet),, TPB, Theory of Planned Behaviour,, VEGD, vegetarian diet,, OMD, omnivorous diet,

## Abstract

There are few studies on what diet mothers following a vegan diet (VD; or strict plant-based diet) choose for their children and how the child’s diet is implemented in everyday life. The present study aimed to explore choices that mothers following a VD make regarding their child’s diet and feeding practices, and what determines these choices. Mothers on a VD whose youngest child was <4 years old were recruited via social media or newsletters about a VD. Participants (N=28) were between 27 and 45 years old and had been adhering to a VD between 0.5 and 23 years. Online semi-structured interviews based on the Theory of Planned Behaviour were audio-recorded and transcribed verbatim. A hybrid thematic analysis approach was used to identify themes that emerged from the data. Mainly driven by ethical considerations of eating animal products, 21 (75%) women chose a VD for their child(ren). When the participant’s partner followed a vegetarian diet (VEGD) or omnivorous diet (OMD), most women chose a VEGD (N=4) or OMD (N=3) for their child as well. Overall, women indicated to make well-considered choices regarding children’s diet and related feeding practices. Determinants for the dietary choice for their children involved various motivations, attitudes, norms, facilitating/hindering factors and knowledge. In conclusion, driven mainly by ethical motivations, most women on a VD chose this diet for their children as well. Despite experiencing several hindering factors and acknowledging the potential negative effects of a VD, mothers seemed to make well-considered choices concerning their child’s diet.

Over the last few years, the interest in a vegan diet (VD) has increased worldwide.^(1)^ A VD (or strict plant-based diet) entails a wide variety of foods but excludes animal-based products, such as meat, dairy, eggs, and honey.^(2)^ Reasons for a VD are often animal-, self- (e.g., health, taste), or environmental-related.^(3,4)^ In the Netherlands, approximately 150.000 people had a vegan lifestyle in 2020 (i.e., 0.86% of the Dutch population).^(5)^ This number will likely increase in the upcoming years as health organisations recommend the general population to increase plant-based nutrition.^(6)^ A VD is associated with lower risks on chronic diseases, such as cardiovascular diseases and cancers.^(7,8)^ However, there is a risk for certain nutritional insufficiencies, such as vitamin B12, zinc, calcium, and selenium.^(9)^

Early childhood is a period that requires adequate nutrition for proper growth, (neuro)development, and health, now and later in life.^(10)^ Parents play a crucial role in determining a child’s dietary intake.^(11)^ While many vegans are young women, it is unknown how many children are raised on a VD, but this number is likely to increase in line with the increasing numbers of adults adhering to a VD.^(1,4)^ Although some healthcare associations discourage a VD for children,^(12,13)^ the Academy of Nutrition and Dietetics states that a well-planned VD is suitable for all life stages, including pregnancy, lactation, infancy, and childhood.^(14)^ Mothers following a VD have been found to produce nutritionally adequate breast milk, but studies showed lower amounts of certain fatty acids.^(15)^ While most infant formulas contain cow milk, soy- or rice-protein-based formulas seem suitable for a VD, and result in normal growth, although more (longitudinal) research is needed.^(16–19)^

Even though a VD in young children is likely to lead to normal growth and might lower the risk on for example overweight, concerns exist as evidence on health outcomes is limited^(20–23)^ and contradicting.^(14,20)^ Parents should provide food with good sources of, among others, vitamin B12, vitamin D, calcium and omega-3, and supplement these if necessary.^(20,21)^ In addition, parents whose children are on a VD are often advised to consult a professional (e.g., a nutritionist or dietitian), especially for recommendations for supplements and introducing potentially allergizing non-vegan foods (e.g., eggs, milk).^(24,25)^ However, health professionals generally involved in early life, such as child health centres’ nurses and paediatricians, are not always informed by parents about the child’s VD.^(26,27)^ Reasons behind this include that parents do not consider this information as important, or fear judgement by the health professional.^(26)^ Additionally, parents often feel that health professionals’ advice about vegetarian or vegan weaning is insufficient.^(26,27)^ Health professionals themselves also often indicate have inadequate knowledge about the potential impact of a VD throughout the life cycle.^(13,28,29)^

Although it is likely that an increasing number of children will be raised on a VD in the upcoming years, only one previous, small sampled study examined which diet is chosen for children of mothers following a VD.^(30)^ Of the eight included mothers, six chose a VD for their child(ren). Moreover, limited research exists on how parents make this choice and implement a child’s VD in everyday life. Bivi *et al*.^(26)^ found that approximately 90% of the children following a VD consumed food from all six relevant VD food groups (fruits, vegetables, grains, protein foods, nuts/seeds, fats/oils) and received a B12 supplement. Also, 66.4% were rarely or never given ready-made meals and 98% of the parents would allow their child to eat animal-based food if they would want to. Parents’ motivation for choosing a VD for their children included health or ethical reasons (75.5%), environmental reasons (5.7%), or no specific reason (16.5%).^(26)^ Other facilitating or complicating factors could potentially influence this choice, such as the unavailability of vegan meals at schools and with family or friends.^(26)^ Moreover, parental concerns (e.g., about the food’s nutritional values) could influence the decision to raise a child on a VD. Additionally, as seen in adults, other factors could influence the decision to follow a more plant-based (including vegan) diet, such as the availability and perceived costliness of products and the perceived difficulty of preparing a plant-based meal.^(31)^ However, research on whether these factors also determine parental choices to adopt a VD for their child and related feeding practices is lacking.

With the increasing popularity of a VD^(1)^ and an expected increase of children following this diet, it is important to know what choices parents on a VD make regarding their child’s diet, as this may influence the child’s health and development. Furthermore, it is relevant to gain understanding about socio-psychological and behavioural determinants of choosing a VD, as these aspects are also understudied. Understanding parental motives and determinants of their choices might lead to better support for parents who adopt this diet.

This study aims to examine the following research questions: ‘What choices do mothers following a VD make regarding the diet of their young child and related feeding practices?’ and ‘What determines these choices?’. The determinants are investigated based on the Theory of Planned Behaviour (TPB).^(32)^ In the TPB, the behaviour of (not) adopting a VD for a child by parents and related feeding practices is assumed to be determined by their intention, which is a function of attitude, subjective norm, and perceived behaviour control. Attitude, subjective norm and perceived behaviour control have previously been shown to be good predictors of intention to adopt a more plant-based diet among young adults.^(33)^ In turn, knowledge on vegan feeding practices is a determinant influencing attitude, subjective norm and perceived behaviour control.^(32,34)^

## Method

### Study design

The present study was a cross-sectional qualitative study with a theoretical exploratory approach. Semi-structured interviews with a structured interview guide were conducted among women following a VD, having a young child <4 years old.

### Participants

Dutch or English-speaking mothers (youngest child <4 years old) following a VD were eligible to participate in this study. In accordance with earlier research, a VD, also called a strict plant-based diet, was defined as consuming products of animal origin (e.g., meat, dairy, eggs, honey) less than once a month.^(2)^ Due to participants’ potential recall bias to infanthood, only mothers with a youngest child younger than four years were included. Participants were recruited between March and May 2023 via posts on Facebook pages focusing on a VD and parenthood and a newsletter of the Dutch association for veganism,^(35)^ until data saturation. Interested women were sent an information letter including the aim and procedures of the study and participant’s rights. After deciding to participate in the study, an interview was planned in April or May 2023. No incentives were offered to participants.

### Data collection instruments and procedures

Individual interviews of approximately one hour took place online via video-conferencing software (Zoom) and were audio recorded with participants’ permission. Two researchers (JP and AA) independently conducted interviews, but used the same interview procedures by developing and employing the same structured interview guide as well as having intermediate discussions of their experiences during the interviews. Questions were based on the TPB^(32)^ and previous literature on parental choices about a VD for children.^(26,27,36)^ Additionally, motivation for their choice was specifically asked.^(37)^ The interview guide was reviewed by project members with scientific expertise on a VD, pregnancy, (determinants of) nutritional behaviour and parenting practices (DM and JG). The interview guide (see Supplementary Material 1) was part of a larger protocol, also covering topics around a VD during pregnancy, but this was outside the scope of the current study.

After explaining the aim of the research and participants’ rights, informed consent was provided by all participants and audio recorded. Thereafter, several questions regarding demographics and background were asked, such as age, educational level, residential area (urban/rural), number and age of children, and whether their partner (if applicable) followed a VD as well. Then, participants were asked about the general choices they made for their child’s diet and related feeding practices (e.g., with regard to VD or non-VD, breastfeeding, infant formula, complementary feeding, consulting a professional, and supplements) and motivations for these choices.

Next, specific questions about determinants of the choice for the child’s diet were asked, based on a theoretical framework on the basis of the TPB. Questions related to intention (i.e., likelihood of performing a behaviour) were about how participants expected to implement (vegan) feeding practices for their child in daily life before they took place (mostly during pregnancy). For attitude (i.e., positive/negative evaluation of a behaviour), participants were asked how they evaluated the healthiness of a VD for their child and the advantages and disadvantages they experienced. For subjective norm (i.e., perception of how relevant others value a behaviour and motivation to comply to this), questions were about how participants perceived how relevant others, such as family, friends, healthcare professionals (i.e., child health centres’ nurses and paediatricians) value a VD for their child. Also, participants were asked to evaluate these opinions and their influence on their decision-making. Perceived behaviour control (i.e., perceived capabilities for a behaviour in light of facilitators and hinderers) questions were about which factors facilitated or complicated raising a child on a VD. Participants were also asked how they evaluated their knowledge of vegan feeding and how they acquired this knowledge. Furthermore, for the present study, the TPB was extended with motivation (based on the Self Determination Theory^(37)^) to examine what motivates participants in their child’s diet choice. Therefore, participants were asked about their main motivation for the decision to (not) raise their child on a VD.

### Data processing and analysis

Interviews were transcribed verbatim, pseudonymized and repeatedly read to gain an understanding of topics. A hybrid thematic analysis was used in which constructs (i.e., attitudes) derived from the theoretical framework were used in a deductive approach combined with new emerging themes in an inductive approach.^(38)^ Three researchers (JP, AA and JG) created a coding tree (code name, definition, transcript example, see Supplementary Material 2) and two of them (JP and AA) independently coded 10% (i.e., three of the transcripts) using NVivo software (version 12, QSR International, Melbourne, Australia^(39)^). The resulting Cohen’s kappa coefficient of the inter-rater reliability analysis was 0.91. As this was sufficiently reliable (cut-off point 0.80^(40)^), one of both researchers (either JP or AA) coded each of the remaining interviews. During coding, an iterative process took place in which codes were reduced to subthemes, themes and corresponding theoretical constructs. Then, constructs and themes across transcripts were compared and interpreted. The analysis mainly focused on children <4 years old, but relevant comparisons with older siblings were considered.

## Results

In total, 28 mothers participated (Table 1). The participants’ age varied between 27 and 45 years old and all were highly educated (i.e., higher vocational education or university). The duration of following a VD ranged between 0.5 and 23 years. All women had a partner, of whom 18 followed a VD, 2 a vegetarian diet (VEGD) and 8 an omnivorous diet (OMD). Their children’s age ranged between 2 months and 9 years old, but results focused on children <4 years old.

**Table 1.** Socio-demographic characteristics of the sample (N=28)

Participant	Age (years)	Residential area	Duration VD (years)	Diet choice partner	Age of children	Diet choice child(ren)
1	45	Urban	6	VEGD	3, 8 and 9 years	VEGD
2	33	Urban	5	VD	1 and 2 years	VD
3	33	Rural	7	VD	2 years	VD
4	35	Urban	4	OMD	1 and 4 years	OMD
5	35	Urban	0.5	OMD	7 months, 3 and 6 years	OMD
6	33	Urban	6	VD	2 years and pregnant	VD
7	37	Urban	8	VD	1 and 4 years	VD
8	36	Urban	6	VD	2 and 6 years	VD
9	39	Urban	6	VD	2 and 5 years	VD
10	31	Urban	4	VD	10 months	VD
11	36	Urban	7	VD	1 and 4 years	VD
12	32	Rural	5	VD	11 months and pregnant	VD
13	39	Urban	8	VEGD	1 and 4 years	VEGD
14	36	Urban	9	VD	3 and 5 years	VD
15	33	Urban	1	VD	1 year	VD
16	40	Rural	2	OMD	2 and 6 years	OMD
17	36	Urban	7	VD	2 years	VD
18	39	Urban	5	VD	4 months	VD (intention)
19	32	Urban	5	VD	1 year	VD
20	36	Rural	5	VD	10 months	VD
21	43	Urban	6	VD	2 years	VD
22	40	Urban	7	OMD	2 and 4 years	VD
23	27	Rural	4	VD	3 months	VD (intention)
24	31	Rural	6	OMD	2 and 4 years	VD
25	32	Rural	4	OMD	2 months	VEGD (intention)
26	28	Rural	3	VD	1 year	VD
27	33	Urban	2	OMD	10 months	VEGD
28	41	Urban	23	OMD	2 and 4 years	VD

VD, Vegan diet (i.e., strict plant-based diet); VEGD, Vegetarian diet; OMD, Omnivorous diet.

### Diet choice and feeding practices

#### Diet choice child

Of all women, 75% (N = 21) chose a VD for their child(ren), 14% (N = 4) chose a VEGD, and 11% (N = 3) chose an OMD. This was mostly in accordance with the partner’s diet choice, except for five couples. Three couples chose to raise their child(ren) on a VD, or two couples chose a VEGD, while the partner followed an OMD. However, these partners often consumed non-vegan products in very limited amounts or only outside the home environment: *“We all eat vegan at home, but he sometimes eats meat outside the house, but actually less and less.” (P24)*. In line with this, the children on a VEGD or an OMD also consumed mostly vegan food, with a limited intake of non-vegan products. All women described that the child(ren)’s diet choice was made together with their partner and mostly before or during pregnancy. One participant became vegan at 6 months of pregnancy and explained that the intended choice for their child’s diet then also switched from vegetarian to vegan.

Although parents decided on the child’s initial diet, many women stated that their child(ren) would be free to decide on their own diet at an older age. Some women did not want to let their child(ren)’s diet contribute to any animal suffering of which the child is not yet aware of: *“It’s just such a… sweet, innocent child and then to force a child to contribute to something she may not like, is not okay with me.” (P3)*. A few women described that it is impossible to be in charge of their child(ren)’s diet forever: *“I have to realize that I can’t …[for] his whole life, have that control over it [child’s diet] and that I can really only convey to him… our values and norms.” (P16)*. The majority wanted to inform their child(ren) about the origin and industry of animal-based products when they are older: *“I would like him to know how, what is behind it, how the meat industry works and what alternatives are.” (P12)*. Some stated that it would please them if their child(ren) would eventually consciously choose a VD themselves as well.

#### Breastfeeding/infant formula

Most children were breastfed, and breastfeeding duration ranged between 4.5 months and 8 years. Two children were exclusively fed with plant-based infant formula, and some children got formula after breastfeeding ended or a combination of breastfeeding and formula. Of the children that were raised on a VD and were given infant formula, seven nonetheless received cow milk-based formula and five used plant-based formula. One woman intended to use rice-based formula, but the child did not like it, resulting in switching to cow milk-based formula. Other reasons for using non-vegan types of formula despite an intended VD were poor availability of vegan formula in the Netherlands, worries about the nutritional completeness of vegan formula, and hospitalisation where only non-vegan formula was available. Some breastfeeding women used additional supplements during lactation or a higher dose of their usual supplements, sometimes explaining this would reach their child through the breastfeeding: *“I took a very high dosage [of supplements]. That is from a study by La Leche League. They had a publication about that if you take a very high dosage yourself… then you don’t have to give your child vitamins, because then it ends up in the breast milk.” (P10)*. Most children were given vitamin K and D from birth onwards. Some women indicated they ate ‘extra healthy’ and/or a higher quantity, to produce ‘good quality’ milk: *“I did eat more when I was breastfeeding, but also, yes very consciously. I wanted to eat… as healthily as possible, [to make sure] that I produced very healthy milk.” (P12)*.

#### Potential allergizing non-vegan food

Most women who chose a VD for their child(ren), gave some potential allergizing non-vegan foods (e.g., eggs, milk) to prevent food allergies: *“We did feed an egg a few times. We have, but he is horrified by it. In principle, I also plan to give that [eggs] a few times because it is… an allergen.” (P15).* Some explicitly stated that they did not want to cause allergies in their offspring due to their choices: *“I sometimes bought eggs… I want to prevent… that he could possibly develop an egg allergy later… because I decided not to offer it during the very early life stages.” (P6)*. A few women did not want to introduce these potentially allergizing non-vegan foods, even when advised by health professionals, as this was against their motivation for a VD: *“Even if the health center said: you now have to introduce eggs… to prevent allergies… We [partner and participant] have discussed with each other… they are vegan, are you going to introduce it anyway? If so, if they want to eat it themselves later, at least they will not be allergic to it. But we were like:… we’re so convinced that it [egg] is not part of a… diet that’s best for the world, so then we’re not going to teach her that.” (P8).*

#### Current diet child

Most women described that their child(ren) had a varied VD, sometimes focusing on whole foods: *“We try to…make sure he gets enough green leafy vegetables and then supplement with chickpeas or tofu or lentils, and just make it a bit of a balanced meal in that way.” (P12).* Most women provided their child(ren) with dairy substitutes and meat/fish substitutes. Some perceived certain (processed) vegan/vegetarian meat substitutes as too salty and therefore limited these, focusing on tofu/tempeh/pulses instead: *“I do try to provide as many unprocessed meat replacers as possible, so just tofu and tempeh actually.” (P7)*. A few women indicated that they consciously named substituting products differently than the original products to clarify to their child(ren) that it was vegan: *“We do try, if we have some replacement chicken pieces [vegan chicken-like product] or something, that we don’t call it chicken. But that we say: oh, they are filet pieces or so.” (P17)*. Most women were or intended to be less strict with the child(ren)’s VD when outside the home environment (e.g., with family/play mates/childcare/restaurants), but drew the line at vegetarian: “*We notice that [asking to prepare] vegan food, then you just ask quite a bit of people of course. So, for example at childcare or for friends, we say: vegetarian.” (P7).* The children on a VEGD/OMD in the current study often consumed a vegan diner: *“I’m the cook at home, so we eat for the most part, yes at least dinner is always vegan. Rarely with something extra, for example some pieces of mozzarella… but yes 9 out of 10 times it’s vegan. But the rest of the family sometimes eats something different with their bread, so they sometimes eat cheese.” (P1)*. All women gave or intended to give vitamin D and B12 supplements (or a multivitamin), mostly from the age of 6 months onwards. Additionally, vegan omega 3 supplements were provided by eleven women and iodine by three. All children following a VEGD consumed vitamin D and sometimes B12, multivitamins and vegan omega 3. Children on an OMD got vitamin D and/or multivitamins.

Many women indicated to only go for additional blood checks because of the VD when the child has complaints (e.g., tired, stunted growth) as they perceived it as very invasive. Nine women described that they might do it in the future: *“Once in a while, I like to do that, to just see that… it’s really okay for you [child] how we eat and you don’t lack anything.” (P11)*. One woman went for a blood check for nutritional deficiencies with their older child (>4 years old).

### Determinants of choice for child’s diet

#### Motivation

Although most women mentioned several motivations, ethical consideration of eating animal products was mentioned by the majority of the women as the main motivation for raising their child(ren) on a VD, followed by climate/future of the child on earth and health: *“Look, it’s all very much mixed up now… the environment… and also health. But I think even if that didn’t play a part, I would still do it [chose a VD], if only for the animal suffering.” (P11)*. Other mentioned motivations (in order of frequent to less frequent) were odd feelings of buying animal-based products when not consuming them herself, providing certain norms and values (e.g., respect everything alive), and the conviction that children naturally do not want to eat animals. All women choosing a VD for their child(ren) stated that their motivation for following a VD was the same as for their child(ren)’s diet choice. One woman explained that her personal motivation for a VD became even stronger reflected in the motivation for the child’s diet choice during pregnancy: *“That’s actually exactly what the dairy industry does too: they take the babies away from the mother [cow] to get milk from them because she’s been pregnant, so I thought it was pretty obvious I wasn’t going to do that.” (P2)*.

Four women chose a VEGD for their child(ren) mainly for practical reasons, as their partner followed a VEGD: *“We simply said: we will continue to eat as we do, so we eat vegan in the evening… but my boyfriend, for example, does have cheese on bread… he [the child] can have that too.” (P18)*. Three women raised their children on an OMD, mainly because of their partner’s OMD and/or not wanting to restrict their partner and children: *“Of course, it’s partly because we already have a mixed [VD/OMD] household… and also, give my boyfriend the opportunity to just go get a nice chicken snack with the kids… he also gets a lot of pleasure out of that.” (P4)*.

#### Attitude

The participants’ attitude (perceived advantages and disadvantages) towards a VD for children are shown in Table 2. Many women emphasised the healthiness and completeness of a VD. Women raising their child(ren) on a VEGD or OMD were also generally positive or neutral about a VD’s healthiness for children, but some mentioned awareness of adequate intake of various nutrients.

**Table 2.** Participants’ attitude (perceived advantages/disadvantages) towards a vegan diet for children according to women who choose this diet for their child(ren) (N = 21)

Advantages VD for children	Example citation
Healthier nutrient composition (consuming more fibre and less fat/cholesterol/salt)	*“I do think that if you eat meat or cheese, then your food is much fatter and saltier.” (P9)*
Higher preference for vegetables/fruit	*“The great thing is that they [her children] actually eat everything… they are really good eaters. They eat a lot of fruit and vegetables.” (P24)*
Less sickness	*“What I do notice, and I’m not a health specialist, so I don’t dare to attribute that 100% to that [VD], but my children are really considerably less sick than literally everyone around us.” (P7)*
Consuming less pesticides and antibiotics	*“I think it is an advantage that you ingest much less bio accumulative toxic substances, because… the world is quite toxic these days. It also accumulates in animals… If you eat those animals, you get that in higher concentrations than if you only eat the plant.” (P10)*

Disadvantages VD for children	Example citation
Concerns about nutritional adequacy of the diet	*“Well, for me it’s a bit of a fear that they will get everything in terms of nutrients [in their VD].” (P28)*
Consciousness about diet is required	*“You just have to be careful that you always take that B12 every day and that you just have a diverse eating pattern.” (P2)*
Child might become an outsider	*“I think it might be difficult that she is different from others… that she experiences it that way.” (P19)*

VD, Vegan diet (i.e., strict plant-based diet).

#### Subjective norm

Some women only experienced neutral or positive opinions from people in their environment (e.g., relatives, friends, colleagues, health professionals) about raising their child(ren) on a VD: *“He also happened to be in the hospital recently for some tests and then I do indicate that he eats plant-based, but there is really no response [from health care professionals]. I think it’s just becoming increasingly normal.” (P15)*. Others experienced that some relatives, friends or colleagues did not understand their choices (at first), struggled with it or asked critical questions (e.g., about deficits): *“I sometimes received indirect nice questions, but [that were] actually a judging question… like: but isn’t he lacking something or? And then I had to refute.” (P2)*. Most women indicated that they could easily explain their choices to others, but some found others’ opinions tiring or strange: *“When I just gave birth I found it quite… tiring always having to defend myself. That’s just not fun.” (P3)*. Sometimes women experienced that relatives or colleagues perceived their choices as sad or imposing, or they experienced a stigma that raising your child on a VD is a drastic choice. However, women perceived that all parents, vegan and non-vegan alike, are responsible for their child(ren)’s diet choices or that a VD is the most natural decision for their child: *“Then I think: you also decide for your child that you push meat into her mouth… if you show a child how that animal was slaughtered, that child would not say: ‘yes, nice, give it to me’.” (P23).*

Five women did not inform the child health centre (providing regular health checkups for young children) about raising the child on a VD, because they either perceived the child health centre’s knowledge of a VD as inadequate or the health care providers did not inquire about it: *“They just have a kind of general knowledge … If you’re really going to talk about this [VD] specifically… then I now know that I simply have much more knowledge than they have.” (P7)*. When informed, child health centres nurses or physicians generally did not react negatively, but advised introducing potential allergizing non-vegan foods or consulting a dietitian. Four women stated that they received some criticising questions from the child health centre about the VD as the child was somewhat small for their age.

If their child would ask to taste non-vegan products, most women would or did allow them to taste. Some would find it difficult, and preferably provided vegan alternatives or explained the VD choice to the child: *“I am not forbidding my children, so if my son or daughter later says: I still want to eat meat or milk, then he or she may do so, but I will provide him with the correct information. So, at the age when he can make that choice, which he can’t do yet because he’s 2.5.” (P2)*. Some mothers indicated that older siblings were already aware of their VD and indicated themselves that they did not want to eat animal-based products: *“She went to grandpa and grandma and were going to eat fish and then she herself said ‘well, I think a fish should swim’.” (P7)*.

#### Perceived behaviour control

Women raising their child(ren) on a VD did not perceive this to be difficult to implement this diet in daily practice, especially at home. Various facilitating and/or hindering factors were mentioned of which (un)supportive social environments and little knowledge among healthcare professionals were most frequently mentioned (Table 3). Some women choosing a VEGD or OMD for their child(ren) stated that the ease of raising a child on a VD is especially influenced by factors outside the home environment (e.g., eating with others, others’ opinions).

**Table 3.** Perceived behaviour control (facilitating/hindering factors) for raising children on a vegan diet according to women who choose this diet for their child(ren) (N = 21)

Facilitating factors^a^	Example citation
Supportive family/friends	*“When we eat with family, they usually also have something plant-based, so our daughter can get that too.” (P18)*
Availability of vegan alternatives in supermarkets	*“It is easier that there is now much more available and that it is very well indicated on products.” (P22)*
Living in a city instead of a village	*“My children always drink oat milk… outside the Randstad [large urban region in the Netherlands], they only have soy milk. They are 15 years behind.” (P9)*
Supportive childcare	*“It was a more natural childcare… they already had a lot of vegetarian products… they just switched to a brand… which was already vegan anyway. They handled that so well.” (P14)*
Enjoy cooking/ having cooking skills	*“We make all the dressings and all the sauces ourselves and I get that, you really have to love cooking, because it’s really… a lot of work, especially with a toddler and preschooler hanging on your leg.” (P9)*
Affordability of vegan meals	*“A plant-based diet does not have to be more expensive than an omnivorous diet… legumes, pasta, rice, vegetables, you should also eat all of that in an omnivorous diet and certainly legumes and tofu are not more expensive than meat.” (P3)*
Partner also follows a VD or supports it	*“I am very happy that my husband is also with me [also follows VD], because you also see couples of which one, often the woman, eats plant-based and the partner says: yes, I just want to continue eating my meat… then it is difficult if a child sees that, then you give a certain example.” (P9)*
Positive societal norm regarding a VD	*“I think, it [VD] is just becoming more and more normal.” (P15)*

Hindering factors^a^	Example citation
Little knowledge of others (e.g., among health professionals)	*“It might be easier if indeed a health center just knew: you can raise children very well on a vegan diet.” (P14)*
Negative opinions from the social environment	*“You just get questions about that [raising child on a VD] and you always have to kind of keep it in mind, that you always… have to explain it a lot.” (P14)*
Higher cost of vegan alternatives compared to animal-based	*“We can bear that [cost of vegan products], but I can imagine that if you don’t have that much to spend, it is sometimes quite difficult to maintain such a diet, because they are generally quite expensive products.” (P11)*
Eating in public places	*“It’s just a shame that if you go to a trip, say a museum… that you just know in advance, there’s nothing [vegan food] there.” (P14)*
Following a VD when abroad	*“We no longer go on holiday to France, because it [finding vegan food] is very difficult there.” (P20)*
Unsupportive family/friends	*“My parents babysit for two days… and when she is with them and they go to the bakery… and she gets a cheese snack… They know I’d rather not have it, but I think the memories she makes… are also very important.” (P3)*
Low availability of vegan infant formula in the Netherlands	*“At the time, I had serious doubts about using soy or rice-based [infant formula]… but that was so poorly available… it was temporarily not available in the Netherlands at all.” (P7)*
Unsupportive childcare	*“At the childcare, basically everything they eat is animal-based, so horrible.” (P12)*
Difference between vegan/non-vegan food is unclear for child	*“Young children don’t understand that [whether a product is animal-based or plant-based] yet. I also make [vegan] sausage rolls with her… If she sees another child eating a sausage roll, she’s not like: oh, that’s a dead animal” (P3)*
Politics/lobby	*“I think one of the main things that makes it difficult for the transition to vegan is just the lobby. It is for a good reason [because of the dairy lobby] that the oat milk with VAT [in the Netherlands] will now become super expensive and then chocolate milk, which contains sugar, will not receive a sugar tax.” (P2)*
Eating with playmates/birthday parties	*“What seems a bit more difficult for me… at a certain point when he goes to children’s parties or plays with friends.” (P15)*
Little choice in vegan ready-made infant food	*“We searched all the shops… then you come home and you have 4 types of [vegan] flavors, that’s it.” (P17)*
Going to school in the future	*“The difficult things are of course, if there is a school trip and lunch is provided there.” (P7)*

VD, Vegan diet (i.e., strict plant-based diet).

a The table shows the facilitating and hindering factors ranked from most to least frequently mentioned by women.

#### Knowledge

Women were relatively confident in their knowledge about a VD and raising their child on a VD. It was often indicated that through the years of following a VD, women had educated themselves: *“We are quite investigative… we don’t assume anything, we find it all out, scientifically based.” (P2)*. For their child(ren)’s diet, most women used various information sources such as books, social media, websites, scientific publications or people around them (e.g., partner, colleagues). Some indicated that it could be difficult to find reliable (non-contradicting) information. The majority of the women consulted or intended to consult a professional about their child’s VD to obtain knowledge, getting confirmation about the current way of feeding, because their partner or family wanted it or as it was advised by the child health center: *“Once my first child was born and… about 1.5 or 2 years old… my mother was quite critical about the fact that we eat plant-based and also have a growing child [who eats] plant-based… then I actually decided to talk to a plant-based dietician to kind of shut her [my mother] up.” (P7)*. All women chose dietitians specialised in a VD, sometimes motivated by stating that general dietitians lack knowledge about a VD.

## Discussion

The present study aimed to explore the choices mothers following a VD make regarding the diet of their child(ren), related feeding practices, and what determines these choices, based on the TPB.^(32)^ The results show that parents decided together on the child’s diet, and most frequently, a VD was chosen for children, primarily for ethical reasons. Some children were raised on a VEGD or OMD, most often when the participant’s partner consumed animal-based products. Non-vegan products would, in that case, be available in the household or provided by the fathers, to which these mothers did not oppose. A non-VD for the child was perceived as most convenient and practical in these specific cases. Mothers also stressed that at a later age, children could decide for themselves if they wanted to (continue to) adhere to a VD.

The results indicated that the choice for a VD was most often driven by motivation. Research has indicated that when motivation is self-determined, in contrast to controlled (e.g., coercion), individuals tend to align attitudes and evaluations of control with their self-determined motives.^(41)^ Indeed, in the current study, despite indicating various disadvantages and hindering factors, women were generally convinced of raising children on a VD, most often motivated by ethical considerations. Earlier research found that ethically motivated vegetarian adults showed stronger adherence and feelings of conviction in their diet than vegetarians motivated by environmental or health reasons.^(42,43)^ Additionally, it is known that vegans show strong identification with their diet (i.e., their VD being part of own values), facilitating dietary adherence.^(44)^ This potentially explains the strong motivational drive of women’s choices in the current study.

Regarding attitude in the TPB,^(32)^ most women in the current study emphasised several benefits of a VD for their child(ren), whereas less disadvantages or potential health concerns were mentioned. Although previous research has shown some benefits of a VD for children,^(14,20,45)^ the long-term risks of a VD for (young) children are understudied.^(20,23,45)^ Therefore, more research is needed before drawing firm conclusions on the (un)healthiness of a VD for children. However, it is reassuring that most women in the current study showed awareness of and made well-considered choices about important advised practices^(21,24,25)^ in relation to their child’s diet, such as introducing potential allergizing non-vegan foods and providing supplements advised for a VD.

Outside the home environment, a child’s VD was often less strictly maintained. This is in accordance with research among vegetarian adults showing dietary deviations from their diet most frequently occur at social occasions.^(46)^ Not all participants were supported in their choice for a VD for their child(ren) by their social environment, as described in previous research.^(26,30)^ Relating to subjective norm in the TPB,^(32)^ many women indicated that the social environment, either being supportive or unsupportive, was an important factor in raising children on a VD. Previous research has reported on general negative attitudes and stigmas perceived by individuals following a VD.^(47)^ In line with this, the current study showed that participants’ social environment often expressed doubts about a VD for children. As a result, women often felt obligated to defend their choices. The above-described uncertainties in evidence of (health) benefits and risks could perhaps contribute to this.

In accordance with earlier research among women following a VD,^(27,48)^ most women in the current study had breastfed their child(ren). Regarding women’s experienced perceived behaviour control for raising children on a VD for those not or partly breastfeeding, low availability of vegan infant formula in the Netherlands was an important hindering factor.^(32)^ Although there have been concerns about vegan formulas’ safety and nutritional value, research has shown its nutritional adequacy, as well as potential sustainability benefits compared to cowmilk-based.^(16,18,19)^ Plant-based (rice or soy) formulas are widely used in other (European) countries, especially for cowmilk-allergic infants.^(18,19)^ However, the only protein sources allowed in infant formula in the Netherlands are cowmilk, goatmilk, and soy, with the latter being only available online.^(49)^

Regarding knowledge as a background determinant in the TPB,^(32,34)^ women generally stated to have sufficient knowledge about raising children on a VD and were able to make well-considered choices. Many participants perceived that knowledge among healthcare professionals about a VD was often insufficient, which sometimes was a reason for parents to not inform professionals about their child’s VD. Previous research has shown similar perceptions and experiences among parents,^(26,27)^ as well as healthcare professionals themselves, indicating to have limited knowledge about a VEGD and VD.^(13,28,29)^ Parents would benefit from professionals having sufficient expertise to assist in raising children on a VD, especially given the trend of and recommendations for increased plant-based nutrition and diets.^(1,6)^ Such expertise can perhaps be concentrated in specialised professionals (e.g., dietitians with specific expertise on VD) to which patients can be referred or other reliable resources.

To our knowledge, the current study is the first that elaborately explored choices of women following a VD make for their child(ren)’s diet. A strength of the current study is the relatively large sample, resulting in data saturation for themes related to a VD among children. However, as a VEGD and OMD was not often chosen for children, saturation was not achieved for corresponding themes. Moreover, paternal perspectives were not included in the current study, although mothers are often the decision-makers of children’s meals.^(50)^ All women in the current study were highly educated, despite earlier studies showing fluctuating prevalence of a VD among different education levels.^(4,26)^ It cannot be ruled out that women’s choices were influenced by their education level and that only women who consciously follow a VD were recruited, as most participants indicated to be well-informed, by educating themselves or by consulting professionals. This potential selection bias is a limitation because a VD requires awareness on dietary intake, and individuals choosing a VD out of less conscious reasons (e.g., following dietary trends) might not be sufficiently aware of that. Recruitment through a newsletter and social-media platforms presumably mainly followed by ‘conscious VD adherers’ (as they have searched for the topic on social media), could have provoked this. However, as a VD requires commitment, it could be assumed that the Netherlands (a country where food is widely available and where various animal-based products are often cheaper than plant-based) has mainly conscious VD followers.

With limited evidence available, more (large-scale) studies are needed about the health implications of a VD among children and the choices parents on a VD make for their offspring. Also, understanding the implementation of a VD for a child through the years is a topic for investigation, as the current study indicated that growing up might involve new challenges in following this diet (e.g., school, friends).

### Conclusion

The present study showed that most women following a VD chose a VD for their child. Despite experiencing several hindering factors and acknowledging potential negative effects of a VD, mothers’ choices were mainly driven by ethical motives. Regarding practices of a VD, women often indicated to make well-considered choices for their child. Future research is needed about dietary decisions parents following a VD make for their child and how a VD evolves when growing up in terms of healthiness and daily life implications.

## Supporting information

Pereboom et al. supplementary material Pereboom et al. supplementary material

## Supplementary material

The supplementary material for this article can be found at https://doi.org/10.1017/jns.2025.14
