# Supplementary material for: Mothers adhering to a vegan diet: feeding practices of their young children and underlying determinants — a qualitative exploration
Source: J Nutr Sci. 2025 Mar 21;14:e26. doi: 10.1017/jns.2025.14 (PMC11950696; doi:10.1017/jns.2025.14)
Supplement: Pereboom et al. supplementary material [file S204867902500014Xsup001.docx]

**Supplementary material 1**

Interview guide parent on a vegan diet with child (younger than 4 years old)

Introduction

1. Interviewer introduces herself
2. Purpose of the study
3. Prompt to start recording
4. Informed consent
   1. Are you sufficiently informed about this study? Was the information letter clear? Do you have any questions?
   2. Do you give permission to participate on this basis?
   3. If there are any questions you don't want to answer, you can indicate that
5. Order of interview: participant characteristics, dietary choices, dietary choices during breastfeeding, dietary choices child, determinants

Demographic characteristics: age, country of birth, place of residence (city/village), level of education, duration of following a vegan diet, first pregnancy or already having child(ren), relationship status. If applicable, partner's diet and age of child(ren).

Diet choice:

1. Since when do you eat vegan?
   1. A specific moment or event?

Diet choice breastfeeding:

1. Did you breastfeed or bottle feed? Why?
   1. If breastfeeding, are you still breastfeeding or bottle feeding? Why?
   2. In case of bottle feeding: what type of bottle feeding do you give/have you given your child (vegan or not)?
   3. Does this differ from what you had in mind prior to the infancy period? (intention)
2. In case of breastfeeding:
   1. How long have you breastfed?
      1. Was this different than planned?
      2. If still breastfeeding, how long do you plan to breastfeed?
      3. Have you ever bottle-fed your child and what type was it (vegan or not)?
3. Did you change your diet when you started breastfeeding? Things added or left out from the diet?
   1. If changes, why?
   2. Is this different from what you had in mind prior to breastfeeding? (intention)
4. Did you start taking supplements when you started breastfeeding? (e.g., vitamin B12 and D)
   1. Does this differ from what you had in mind prior to the infancy period? (intention)

Diet choice child

1. Can you tell something about the choice you made for your child's diet?
   1. For raising child on a vegan diet: are there certain animal-based products you still give and why or why not?
2. Since when do you know that you (don’t) want to raise your child on a vegan diet?
   1. Was there one specific moment or event that made you make this choice?
3. Have you thought long about this choice?
4. Did the dietary choice (vegan, vegetarian, etc.) you made for your child during the pregnancy/infant period differ from the diet your child eventually follows? (intention)
   1. If so, why did you eventually make these changes?

Daily practices

1. When did you introduce solid foods to your child?
2. What did these first bites look like?
   1. During the pregnancy/infant period, did you have the same thoughts about how the introduction of food would go? (intention)
      1. E.g., were you planning not to give any animal-based products to your child, but ended up doing this anyway?
3. What did your child's diet consist of from 6 months?
4. What does your child's diet currently consist of?
5. What do you think about ready-made baby food products from the supermarket. Have you used it and/or are you currently using it? (Also discuss meat substitutes)
6. What does your child eat when outside the home environment? For example, with family, on the road or when playing at a friend's house?
   1. Did you feel the same about this during the pregnancy/infant period? (intention)
   2. Possibly, why did the ideas differ from what you end up doing?
7. What do you think about using supplements for infants/young children? Do you give your child supplements?
   1. B12 if this is not discussed automatically.
   2. Vitamin D if this is not discussed automatically.
   3. From what age/moment?
8. Have you consulted a healthcare professional, such as a dietitian or midwife, about raising your child on a vegan diet?
   1. Did you also plan to do this during your pregnancy? (intention)
   2. Possibly, why did the ideas during pregnancy differ from what you ended up doing?
9. For raising child on a vegan diet: have you told the health center that you want to raise/raise your child on a vegan diet?

Motivation for adopting a vegan diet

1. What is the biggest motivation for you personally to follow a vegan diet?
2. What is the biggest motivation for you personally to (not) raise your child on a vegan diet?

Attitude

1. What advantages do you see in raising a child on a vegan diet?
2. What disadvantages do you see in raising a child on a vegan diet?
3. Do you think a vegan diet is healthy for the fetus/baby/child?
4. What was/is your opinion on the completeness (contains all necessary nutrients) of the vegan diet for:
   1. Babies to first bite (bottle feeding or breastfeeding when mother eats vegan)
   2. Babies from first bite
5. For raising child on a vegan diet: what do you think about regularly having extra health checks (e.g., blood tests) done on your baby/child because of the vegan diet?

Subjective Norm

1. What are the opinions of others around you about raising a child on a vegan diet?
   1. Family
   2. Friends
   3. Healthcare professionals (e.g., obstetrician, gynecologist, dietitian)
   4. Social media
   5. Health center
   6. Possibly, childcare
2. What do you think of these opinions?
3. Do these opinions influence the choice of a (non) vegan diet for your child and in what way?
4. Has your child ever indicated that he or she wanted to eat an animal-based product?
   1. How did you deal with this (allow/deny)?

Perceived Behavior Control

1. To what extent do you think you can raise your child on a vegan diet?
2. What factors do you think make it easier to raise your child on a vegan diet? (e.g., opinions of others (social), availability of products (physical) costs of vegan alternatives (economic), childcare policy (political))
3. What factors do you think make it more difficult to raise your child on a vegan diet? (e.g., opinions of others (social), availability of products (physical) costs of vegan alternatives (economic), childcare policy (political))

Knowledge

1. Do you think you have enough knowledge about raising a child on a vegan diet?
   1. Infant formula
   2. Complementary foods from 6 months
2. What resources did you use for learning about raising a child on a vegan diet?
   1. Friends
   2. Family
   3. Social media
   4. Books
   5. Healthcare professionals (e.g., obstetricians, gynecologists, dietitians, nutritionists)
   6. Scientific resources

Finishing interview

1. Do you have anything to add to the conversation we had? Are there things not discussed that you would like to discuss?
2. Do you have any other questions or comments for me?
3. May we approach you for possible future research?
4. Thank for participation

**Supplementary material 2**

**Coding tree with definitions and example citations**

| **Code** | **Definition** | **Example citation** |
| --- | --- | --- |
| **Demographic characteristics** | | |
| Country of birth | Wat is the country of birth of the participant | *“I was born in the Netherlands.” (P1)* |
| City or village | Does the participant live in a rural or urban area | *“I live in X [Dutch city].” (P27)* |
| Educational level | What is the highest educational level of the participant | *“I have a university education.” (P1)* |
| Partner | Does the participant have a partner | *“I have a husband.” (P24)* |
| Number of children | Number of children of the participant | *"I have one daughter." (P26)* |
| Age children | What is the age of the child(ren) of the participant | *“We have a two-year-old son.” (P21)* |
| Growth child omnivorous | Any statements on the growth of the child on an OMD | *“Yes, they were both a bit on the small side.” (P4)* |
| Growth child vegan | Any statements on the growth of the child on a VD diet | *“Well, look my daughter is super healthy, super happy, super fit, she's growing really well, she's developing really well.” (P10)* |
| Growth child vegetarian | Any statements on the growth of the child on a VEGD | *“They are for the height of an Indonesian father and I am not yet 1.60 meters so, well, they are above our [growth] curve, but below the Dutch curves. It's just totally fine.” (P13)* |
| **Diet choice and motivation** | | |
| **Diet choice** | The diet choice of the participant that currently follows a VD | |
| Own diet, strictness, whole food, organic | What does the VD of the participant look like in terms of strictness in excluding non-vegan products, following the whole food principles or consuming (only) organic products | *“I eat strictly plant-based. I think it happened once in a restaurant that there accidentally was egg in it, but then I just ate it. Not consciously, as long as I have control over it, I don't eat it.” (P10)* |
| How long vegan | For how long follows the participant a VD | *“I have been eating completely vegan for 7 or 8 years now.” (P3)* |
| Process or 1 moment | Was the transition to a VD a process or one moment for the participant | *“That [vegan diet] grew out of vegetarianism a bit for me. Once as a child I thought it was very sad and at a certain point… it also became animal suffering. Eventually, I thought vegan was the only option there was actually.” (P4)* |
| Substituting products of animal origin | How did the participant substitute products of animal origin when the transition to a VD was made | *I: “Do you use a lot of substitutes… thus dairy substitutes and meat substitutes?”*  *P: “Yes… soy yogurt, coconut yogurt, almond yogurt every day… I try not to always make a burger or something on the side for dinner, but… on average five evenings out of seven a week, something like a [meat] substitute is prepared for dinner.” (27)* |
| **Diet choice partner** | The diet choice of the partner is a VD | *“We both actually eat… completely vegan.” (P9)* |
| Diet choice partner when not vegan | What is de diet choice of the partner when this is not a VD | *“He eats [a vegan] dinner with us, but generally, he eats everything.” (P27)* |
| Diet choice process partner | What is the process of the partner of starting to follow the current diet | *“At a certain point… he has also just made that transition, but just a little longer, a little slower. Because he first thought of: yes, if I am on my way and I want to eat something and there is nothing [vegan] yes, then I think that I should be able to just get an egg sandwich or something.” (P14)* |
| How long vegan partner | For how long follows the partner a VD | *“And I think he's been eating completely plant-based for about 4 years now.” (P3)* |
| **Diet choice child from age 4** | What choice did the participant made for the diet of their child (≥4) | *“We also raise our children vegan.” (P11)* |
| In case of vegan, any remaining products from animal origin | In case of a VD choice for the child (≥4), are there any products from animal origin that the child (≥4) still gets of have gotten | *“We did give an egg a few times. We have, but he is horrified by it. In principle, I also plan to give that [eggs] a few times because it is... such an allergen.” (P15)* |
| Intention diet choice child | What is/was the participant's intention of which diet would be chosen for the child (≥4) | *“I think during the pregnancy, we just talked about how we would like to do that [raising child on a VD].” (P8)* |
| **Diet choice child below age 4** | What choice did the participant made for the diet of their child (<4) | *“I find it very difficult to say that they were born vegan, because it is of course a personal choice, but we do raise them with a vegan vision. I think that's a pretty good description.” (P14)* |
| In case of vegan, any remaining products from animal origin | In case of a VD choice for the child (<4), are there any products from animal origin that the child (≥4) still gets of have gotten | *“Even if the health center said: yes, you now have to introduce eggs… to prevent allergies… We have discussed with each other… they are vegan, are you going to introduce it anyway? If so, if they want to eat it themselves later that, at least they're not allergic to it. But we were like: yes, we're so convinced that it [egg] is not part of a... diet that's best for the world, so then we're not going to teach her that or anything.” (P8)* |
| Intention diet choice child | What is/was the participant's intention of which diet would be chosen for the child (<4) | *“I think during the pregnancy, we just talked about how we would like to do that [raising child on a VD].” (P8)* |
| Motivation diet partner when not the same | What is the main motivation of the partner for his or her diet choice when this is not the same motivation as the participant's | *“For my boyfriend it is more, for him health is, I think, the biggest factor. Yes, it is.” (P15)* |
| Motivation diet choice child omnivorous | What is the main motivation of the participant to raise their child(ren) on an OMD | *“Of course, it's partly because we already have a mixed household [father has OMD]. So, for me it's like, they just eat what's there and what they want to eat of it. And then of course, I am always very happy when they say: oh, I like the mama [plant-based] cheese better than the papa [animal-based] cheese haha… And also, give my boyfriend the opportunity to just go get a nice chicken snack with the kids… He also gets a lot of pleasure out of that.” (P4)* |
| Motivation diet choice child vegan | What is the main motivation of the participant to raise their child(ren) on a VD | *“Basically, the same consideration as for ourselves, so health is important, tastiness is important. But we also think it is certainly important that the food pattern does not harm others, including animals.” (P10)* |
| Motivation diet choice child vegetarian | What is the main motivation of the participant to raise their child(ren) on a VEGD | *“I just don't think it's necessary at all for him to eat something that animals have suffered for and I also just really, really don't think he needs it, because there are so many [vegan] alternatives.” (P25)* |
| Motivation vegan | What is the main motivation of the participant to follow a VD | *“The main reason was the ethical aspect and later I also saw that plant-based, or at least [eating] more plant-based food has health benefits and there is now of course a lot of concern about environmental impact…those things together actually make the choice even clearer.” (P18)* |
| **Infant period** | | |
| **Infant period child younger than 4 omnivorous** | Practices during the infant period of a child younger than 4 on an OMD | |
| Breastfeeding child younger than 4 | Is/was the child (<4) on an OMD breastfed and if terminated, how long was the child breastfed | *“I have for a few months. Breastfeeding and partly bottle feeding. I breastfed for a few months and also infant formula.” (P4)* |
| Own diet during breastfeeding | What is/was the participant’s diet during breastfeeding | *“I didn't consciously do anything else because I was breastfeeding.” (P4)* |
| Infant formula child younger than 4 | Is/was the child (<4) on an OMD given infant formula and which type | *“From the very beginning, we have also supplemented the children with regular powdered milk [infant formula], so to speak. So that was always kind of a combination.” (P4)* |
| **Infant period child younger than 4 vegan** | Practices during the infant period of a child younger than 4 on a VD | |
| Breastfeeding child younger than 4 | Is/was the child (<4) on a VD diet breastfed and if terminated, how long was the child breastfed | *“Yes, exclusively breastfed, so she is still breastfed now.” (P3)* |
| Own diet during breastfeeding | What is/was the participant’s diet during breastfeeding | *“I noticed that I needed a lot of energy… even more so than during my pregnancy. I ate a lot while breastfeeding.” (P19)* |
| Infant formula child younger than 4 | Is/was the child (<4) on a VD given infant formula and which type | *“From 6 months, he is now 11 months, we switched to bottle feeding and he gets cow's milk, AH [Albert Heijn, brand] organic, so organic milk.” (P12)* |
| **Infant period child younger than 4 vegetarian** | Practices during the infant period of a child younger than 4 on a VEGD | |
| Breastfeeding child younger than 4 | Is/was the child (<4) on a VEGD breastfed and if terminated, how long was the child breastfed | *“Exclusively breastfed until half a year, because I wanted to, as that is recommended by the World Health Organization.” (P1)* |
| Own diet during breastfeeding | What is/was the participant’s diet during breastfeeding | *“I think [I ate] a little more [food] than during pregnancy… I just notice that I'm hungry more often and I didn't have that during pregnancy, because my belly was full of a baby haha.” (P25)* |
| Infant formula child younger than 4 | Is/was the child (<4) on a VEGD given infant formula and which type | *“No, he was never bottle fed.” (P1)* |
| **Infant period child older than 4 omnivorous** | Practices during the infant period of a child from 4 on an OMD | |
| Breastfeeding child older than 4 | Is/was the child (≥4) on an OMD breastfed and if terminated, how long was the child breastfed | *“Breastfeeding, that was a few months. I think… the first one maybe 6 months.” (P4)* |
| Own diet during breastfeeding | What is/was the participant’s diet during breastfeeding | *“I didn't change anything there [diet during lactation period] except maybe start eating things that I didn't want during my pregnancy, but yes, that wasn't a lot. But I didn't consciously do anything else because she was breastfeeding.” (P4)* |
| Infant formula child younger than 4 | Is/was the child (≥4) on an OMD given infant formula and which type | *“From the very beginning, we have also supplemented the children with regular powdered milk [infant formula], so to speak. So that was always kind of a combination.” (P4)* |
| **Infant period child older than 4 vegan** | Practices during the infant period of a child from 4 on a VD | |
| Breastfeeding child older than 4 | Is/was the child (≥4) on a VD breastfed and if terminated, how long was the child breastfed | *“The first [child] was eventually breastfed for 6 months.” (P7)* |
| Own diet during breastfeeding | What is/was the participant’s diet during breastfeeding | *“I just ate things in the evening too, so I always had nuts on hand. I always ate a handful of nuts.” (P8)* |
| Infant formula child younger than 4 | Is/was the child (≥4) on a VD given infant formula and which type | *“We used Novarice [rice-based formula] from Novalac [brand] as bottle feeding with the eldest.” (P14)* |
| **Infant period child older than 4 vegetarian** | Practices during the infant period of a child from 4 on a VEGD | |
| Breastfeeding child older than 4 | Is/was the child (≥4) on a VEGD breastfed and if terminated, how long was the child breastfed | *“The middle one, who is now 8, has been breastfed for a long time. We stopped on her eighth birthday.” (P1)* |
| Own diet during breastfeeding |  | *“I ate a lot!” (P13)* |
| Infant formula child younger than 4 | Is/was the child (≥4) on a VEGD given infant formula and which type | *“I: Did they get any formula afterwards?”*  *“P: Yes.” (P13)* |
| **Daily diet of the children** | | |
| Availability of products of animal origin at home | The availability of products of animal origin (e.g., meat, dairy, eggs and honey) at home | *“Occasionally they eat meat, but I try to limit it and when I do the shopping, very rarely that I buy anything made of meat and that is really for special days… when someone has a birthday and they ask specifically for something, then they get that, but if I do the shopping… I am not going to think in advance of: oh I'm going to make something with meat.” (P5)* |
| **Diet child under 4** | The diet of a child younger than 4 | |
| Daily diet, variation omnivorous | The general eating pattern and variation of a child (<4) on an OMD | *“The evening meals are mostly vegan, but they actually eat meat once a week and fish once a week, something like that. And also just cow's milk, cheese. The youngest gets cow's milk, cow’s yogurt… and the oldest soya, because he doesn't like the cow yogurt.” (P4)* |
| Daily diet, variation vegan | The general eating pattern and variation of a child (<4) on a VD | *“We try just like I do with myself, make sure he gets enough green leafy vegetables and then supplement with chickpeas or tofu or lentils or, and just make it a bit of a balanced meal in that way.” (P12)* |
| Daily diet, variation vegetarian | The general eating pattern and variation of a child (<4) on a VEGD | *“I'm the cook at home, so we eat for the most part, yes at least dinner is always vegan. Rarely with something extra, for example some pieces of mozzarella or something, but yes 9 out of 10 times it's vegan. But the rest of the family sometimes eats something different on bread, so they sometimes eat cheese… well, that's it actually: milk is no longer in the fridge haha.” (P1)* |
| Eating outside the home environment omnivorous | What does a child (<4) on an OMD eat when eating outside the home environment | |
| Family omnivorous | What does a child (<4) on an OMD eat when eating with family | *“I really like that they can taste everything and we are very free with everything, including sugar for example. Yes, you can, it's all in moderation, but you can really try it all once and if it's a party or we're at grandpa and grandma's or eating out… then we don't make a fuss about it.” (P4)* |
| Parents' friends omnivorous | What does a child (<4) on an OMD eat when eating with friends of the parents | *No citation* |
| Restaurant omnivorous | What does a child (<4) on an OMD eat when eating out | *“Usually then yes, then we take that [ready-made infant food] with us or then we just go somewhere where we know there is a kid's meal.” (P4)* |
| Friends omnivorous | What does a child (<4) on an OMD eat when eating with a play mate | *“I: If your children go to play with friends, you just mentioned that with the childcare and school, then you actually let it [what the child eats] go, if I understood correctly?”*  *“P: Yes, yes.” (P16)* |
| Eating outside the home environment vegan | What does a child (<4) on a VD eat when eating outside the home environment | |
| Family vegan | What does a child (<4) on a VD eat when eating with family | *“Our parents, they just cook plant-based meals for us. Or they make it easy for themselves… just potatoes and vegetables… we can eat that and then just for us a vegan burger.” (P26)* |
| Restaurant vegan | What does a child (<4) on a VD eat when eating out | *“If we really go out for dinner with the three of us… then we often go to a vegan restaurant and then it is all fine anyway. And X [child] likes almost everything and she eats a few bites of everything, so that's very nice.” (P3)* |
| Parents' friends vegan | What does a child (<4) on a VD eat when eating with friends of the parents | *“With friends, I must say, yes very often friends also make sure that they make something vegetarian or vegan.” (P6)* |
| Friends vegan | What does a child (<4) on a VD eat when eating with a play mate | *“We notice that [asking to prepare] vegan food, then you just ask quite a bit of people of course. So, for example at childcare or for friends, we say: vegetarian.” (P7)* |
| Eating outside the home environment vegetarian | What does a child (<4) on a VEGD eat when eating outside the home environment | |
| Family vegetarian | What does a child (<4) on a VEGD eat when eating with family | *“The only thing is that we really pressed the grandpas and grandmas and continue to press the heart, that we really don't want them to eat it [meat/fish]. I know that my mother once secretly let them eat a shrimp, because it looked so nice. And then I say: yes, you know, it won't kill them, but it's not healthy for them, it's not good for them. So, I prefer that we do that consciously and not that you secretly do that.” (P13)* |
| Restaurant vegetarian | What does a child (<4) on a VEGD eat when eating out | *No citation* |
| Parents' friends vegetarian | What does a child (<4) on an OMD eat when eating with friends of the parents | *No citation* |
| Friends vegetarian | What does a child (<4) on a VEGD eat when eating with a play mate | *“I'm just saying [to play mates’ parents]:… well, she does not die from a nut but she doesn't eat animals… and that's what I'm telling and that's actually pretty okay these days.” (P13)* |
| Eating at childcare omnivorous | What does a child (<4) on an OMD eat when eating at childcare | *“For example, she goes to childcare 2.5 days a week and at the moment she only eats there, well, fruit and breastfeeding or bottle feeding.” (P5)* |
| Intention eating at childcare or school later | What is/was the participant's intention of what the child (<4) on an OMD will eat at childcare or school later | *“I think, I'm going to say at the childcare, that she just eats vegetarian. Because I don't really feel the need for her to get cold cuts on bread every time and then start thinking: why don't we have that at home. No… she just won't be given that [at childcare].” (P5)* |
| Eating at childcare vegan | What does a child (<4) on a VD eat when eating at childcare | *“With the first child, I always gave a cup of soy milk for lunch. And now they buy it themselves, because there is another child who drinks that, so now they have it available.” (P22)* |
| Intention eating at childcare or school later | What is/was the participant's intention of what the child (<4) on a VD will eat at childcare or school later | *“She's going to childcare and soon she's going to school… Together, my partner and I, chose not to let her eat anything else then if, for example, they get a [non-vegan] treat in class or if she has a children's birthday party and there's… a whipped cream pie or something else. I really want her to be able to join in with the rest.” (P3)* |
| Eating at childcare vegetarian | What does a child (<4) on a VEGD eat when eating at childcare | *“At the childcare, they also occasionally get milk and well, also yogurt and things like that and I just let that go a bit for now.” (P5)* |
| Intention eating at childcare or school later | What is/was the participant's intention of what the child (<4) on a VEGD will eat at childcare or school later | *“I: Have you already been in contact with childcare, just to see how they feel about it [child’s VEGD]?”*  *“P: No, yes, I registered him, yes. But no contact yet… but I think, because I hear from friends, right, because I also have friends who eat vegetarian, so they know, that it [letting child eat vegetarian at childcare] is possible.” (P25)* |
| What did bites look like omnivorous | How did the first bites of the child (<4) on an OMD look like | *“That was just a mashed pumpkin or, at least it was always vegetables… that was the case in the first two months, really the pureed vegetables… after that, we kind of switched to a kind of mix form that we also just gave whole pieces and sometimes just a mash, sometimes we fed them, sometimes they ate it themselves. We mixed that up a bit.” (P4)* |
| What did bites look like vegan | How did the first bites of the child (<4) a VD look like | *“We followed the Kleintjes method or Rapley, so then you offer whole pieces and then there is an introduction schedule with what they can eat when, with the intestinal development. So, we start, then only started with 6 months, that they can really just sit and then we always just gave those bites, vegetables and at some point, pasta.” (P9)* |
| What did bites look like vegetarian | How did the first bites of the child (<4) a VEGD look like | *“I don't really know very well anymore. I do know that we started with vegetable snacks, because that is a bit more difficult... With the other two [children]… I think something like cauliflower… because it is still a bit sweet.” (P1)* |
| Intention introducing bites omnivorous | What is/was the participant's intention of how the introduction of the first bites will go for a child (<4) on an OMD | *“I really wanted to do that Rapley method. But in that respect, I was flexible again and noticed… the oldest wanted to eat with the spoon herself very quickly, so then… we just gave her the spoon, as a little baby and then she just went for it, to do it herself. And the youngest, he was much more of just give it to me haha.” (P4)* |
| Intention introducing bites vegan | What is/was the participant's intention of how the introduction of the first bites will go for a child (<4) on a VD | *“When we started with solid foods, I really wanted to, do it kind of the Rapley way.” (P3)* |
| Intention introducing bites vegetarian | What is/was the participant's intention of how the introduction of the first bites will go for a child (<4) on a VEGD | *“Now that you mention it, I think I wanted to do more Rapley method with him, so those are bits and pieces, but I don't think it worked that way and then we started doing more mushes.” (P1)* |
| Ready-made products omnivorous | Does the participant use ready-made (baby) products for their child (<4) on an OMD | *“I just have a jar [ready-made infant food] like that sometimes. Not so often such a real evening meal. We have done that sometimes, because you also have like that pure vegetable jars… I have used jars, but they were always vegetarian, so I never used meat variants. And those snacks, I use them very often. Those fruit bags and those, those chips and stuff, those corn nibbles and stuff, we use that a lot.” (P4)* |
| On the go, outdoors omnivorous | Does the participant use ready-made (baby) products for their child (<4) on an OMD when on the go or outside the home environment | *“Usually then yes, then we take that [ready-made infant food] with us or then we just go somewhere where we know there is a kid's meal.” (P4)* |
| Meat and dairy substitutes omnivorous | Does the participant use meat and dairy substitutes for their child (<4) on an OMD | *“We also don't buy that much meat, really those meat replacers that are a bit more expensive. And sometimes there is something on offer and then: oh nice, I'll take that with me. Then we'll try that.” (P4)* |
| Ready-made products vegan | Does the participant use ready-made (baby) products for their child (<4) on a VD | *“Because we didn't give pureed food, we never had those glass food jars [ready-made infant food].” (P26)* |
| On the go, outdoors vegan | Does the participant use ready-made (baby) products for their child (<4) on a VD when on the go or outside the home environment | *“We sometimes have one of those, those squeeze bags, fruit bags, but we really only use them for on the road, when we're in the car or something. Other than that, I just make everything myself.” (P12)* |
| Meat and dairy substitutes vegan | Does the participant use meat and dairy substitutes for their child (<4) on a VD | *“They like tofu, but most meat substitutes… they usually do not like them that much.” (P24)* |
| Ready-made products vegetarian | Does the participant use ready-made (baby) products for their child (<4) on a VEGD | *“Yes, we did then [use ready-made infant food], because it was just really, yes with 3 children, it was quite intense to prepare all things when you are back at work and so on… then I did indeed buy jars [ready-made infant food] for him and he ate them well, so it worked there.” (P1)* |
| On the go, outdoors vegetarian | Does the participant use ready-made (baby) products for their child (<4) on a VEGD when on the go or outside the home environment | *“Yes, more on vacation or something, so. If we then went to a restaurant or something, not very often [used ready-made infant food]. He would eat there. Usually, we ordered for 4 and then he could just take what he liked from us, yes. And yes, more for camping or something, than those jars [ready-made infant food].” (P1)* |
| Meat and dairy substitutes vegetarian | Does the participant use meat and dairy substitutes for their child (<4) on a VEGD | *“Yes, they like that. Fake fish fingers or fake schnitzel, yes, they really like that, for sure.” (P13)* |
| Fussy or easy eater omnivorous | Is the child (<4) on an OMD a fussy or easy eater | *“The eldest is a bit of a picky eater, in fact they both are a bit.” (P4)* |
| Fussy or easy eater vegan | Is the child (<4) on a VD a fussy or easy eater | *“He likes quite a lot so far, but he likes fruit a little less… He really likes vegetables, so that's nice.” (P20)* |
| Fussy or easy eater vegetarian | Is the child (<4) on a VEGD a fussy or easy eater | *“And the youngest, who is a really bad eater anyway.” (P1)* |
| Moment of introducing first bites omnivorous | The moment when parents introduced the first bites to their child (<4) on an OMD | *“Well with 4 months or say, from the moment it was possible.” (P4)* |
| Moment of introducing first bites vegan | The moment when parents introduced the first bites to their child (<4) on an VD | *“I think with 6 months, because we did the Rapley method... and that was possible from the time she could sit and she was already sitting at 5 to 6 months.” (P19)* |
| Moment of introducing first bites vegetarian | The moment when parents introduced the first bites to their child (<4) on a VEGD | *“I wanted to see if we could also postpone those first bites to six months and it seemed that we could, because he didn't need it that much at all. So yes, exclusive breastfeeding for the first six months and then started with the first snacks.” (P1)* |
| **Diet child from 4** | The diet of a child 4 years and older | |
| Daily diet, variation omnivorous | The general eating pattern and variation of a child (≥4) on an OMD | *“When I cook it is completely vegan. Sometimes there is something extra [non-vegan], because we have a fish stall and a chicken stall… then sometimes we get something there, then it just comes on top and when my boyfriend cooks, it is also basically vegan, but then he usually gets something [non-vegan] to go with it.” (P4)* |
| Daily diet, variation vegan | The general eating pattern and variation of a child (≥4) on a VD | *“We always eat oatmeal for breakfast with soy milk and banana in it and sometimes something like raisins or seeds… That is actually 90% of the time the food in the morning, yes or strawberries or berries or, it is, alternating for that matter, but it is basically oatmeal.” (P7)* |
| Daily diet, variation vegetarian | The general eating pattern and variation of a child (≥4) on a VEGD | *“They really eat everything: from my in-laws' Indonesian food, to potatoes, pastas.” (P13)* |
| Eating outside the home environment omnivorous | What does a child (≥4) on an OMD eat when eating outside the home environment | |
| Family omnivorous | What does a child (≥4) on an OMD eat when eating with family | *“I really like that they can taste everything and we are very free with everything, including sugar for example. Yes, you can, it's all in moderation, but you can really try it all once and if it's a party or we're at grandpa and grandma's or eating out… then we don't make a fuss about it.” (P4)* |
| Restaurant vegetarian | What does a child (≥4) on an OMD eat when eating out | *“Usually then yes, then we take that [ready-made infant food] with us or then we just go somewhere where we know there is a kid's meal.” (P4)* |
| Parents' friends omnivorous | What does a child (≥4) on an OMD eat when eating with friends of the parents | *No citation* |
| Friends omnivorous | What does a child (≥4) on an OMD eat when eating with a play mate | *“I: If your children go to play with friends, you just mentioned that with the childcare and school, then you actually let it [what the child eats] go if I understood correctly?”*  *“P: Yes, yes.” (P16)* |
| Eating outside the home environment vegan | What does a child (≥4) on a VD eat when eating outside the home environment | |
| Family vegan | What does a child (≥4) on a VD eat when eating with family | *“Now they just eat what is served and as said, we are just lucky that our family handles that [vegan diet] very well.” (P14)* |
| Restaurant vegan | What does a child (≥4) on a VD eat when eating out | *“When we go out for dinner, what I usually do is just order a basket of bread for them, especially the oldest one, which can have some fries or something or a few bites of tomato soup… things like that.” (P7)* |
| Parents' friends vegan | What does a child (≥4) on a VD eat when eating with friends of the parents | *“Then just say: well, just eat what you eat and then you do it [preparing the meal] separately for them, actually.” (P9)* |
| Friends vegan | What does a child (≥4) on a VD eat when eating with a play mate | *“Fortunately, he [child] has a few friends there, who know very well… how it [VD] works and also at children's parties that we have indicated: we can bring our own [vegan] food. Or it was taken into account. It varies a bit by yes, per parent, where he goes.” (P14)* |
| Eating outside the home environment vegetarian | What does a child (≥4) on a VEGD eat when eating outside the home environment | |
| Family vegetarian | What does a child (≥4) on a VEGD eat when eating with family | *“Well, actually they always eat what is served and if they really don't like it, then I'll make something up.” (P13)* |
| Restaurant vegetarian | What does a child (≥4) on a VEGD eat when eating out | *No citation* |
| Parents' friends vegetarian | What does a child (≥4) on a VEGD eat when eating with friends of the parents | *No citation* |
| Friends vegetarian | What does a child (≥4) on a VEGD eat when eating with a play mate | *“I'm just saying [to play mates’ parents]:… well, she does not die from a nut but she doesn't eat animals… and that's what I'm telling and that's actually pretty okay these days.” (P13)* |
| Eating at childcare or school omnivorous | What does a child (≥4) on an OMD eat when eating at childcare or school | *“The eldest goes to school of course… I texted recently, because then he was going to treat and then I… made bags with sweets and then I texted to the teacher “well for the teachers, I made a plant-based apple-cinnamon cake and vegan sweets for all the children”. I don't know if she took that hint.” (P16)* |
| Eating at childcare or school vegan | What does a child (≥4) on a VD eat when eating at childcare or school | *“At school X [child] does have her treat box, so if there are [non-vegan] treats she can't eat, then I made a box with all kinds of vegan sweets and things she likes.” (P8)* |
| Eating at childcare or school vegetarian | What does a child (≥4) on a VEGD eat when eating at childcare or school | *No citation* |
| What did bites look like omnivorous | How did the first bites of the child (≥4) on an OMD look like | *“That was just a mashed pumpkin or, at least it was always vegetables… that was the case in the first two months, really the pureed vegetables… after that, we kind of switched to a kind of mix form that we also just gave whole pieces and sometimes just a mash, sometimes we fed them, sometimes they ate it themselves. We mixed that up a bit.” (P4)* |
| What did bites look like vegan | How did the first bites of the child (≥4) on a VD look like | *“Oh, I do have to recall very well, I think you start around, between 6 and 8 months, you start with a bit of those practicing snacks… they say: start with vegetables because those are the more difficult flavors, so mashed carrot and sweet potato and spinach, broccoli, things like that. We really started with pure flavors… so not much mixed up yet” (P11)* |
| What did bites look like vegetarian | How did the first bites of the child (≥4) on a VEGD look like | *“Yes, the eldest with 4 to 6 months and just neat and tasty mashed bites, just as it should be. With the first one, you can still do all that neatly.” (P13)* |
| Intention introducing bites omnivorous | What is/was the participant's intention of how the introduction of the first bites will go for a child (≥4) on an OMD | *“I really wanted to do that Rapley method. But in that respect, I was flexible again and noticed… the oldest wanted to eat with the spoon herself very quickly, so then… we just gave her the spoon, as a little baby and then she just went for it, to do it herself. And the youngest, he was much more of just give it to me haha.” (P4)* |
| Intention introducing bites vegan | What is/was the participant's intention of how the introduction of the first bites will go for a child (≥4) on a VD | *No citation* |
| Intention introducing bites vegetarian | What is/was the participant's intention of how the introduction of the first bites will go for a child (≥4) on a VEGD | *No citation* |
| Ready-made products omnivorous | Does the participant use ready-made (baby) products for their child (≥4) on an OMD | *“With my first child, yes, it is maybe also a bit, yes with your first you have well, and more time and it is your first, so you try to be a bit, well more perfect at that. My first one actually didn't have any jars [ready-made infant food] at all.” (P5)* |
| On the go, outdoors omnivorous | Does the participant use ready-made (baby) products for their child (≥4) on an OMD when on the go or outside the home environment | *“Usually then yes, then we take that [ready-made infant food] with us or then we just go somewhere where we know there is a kid's meal.” (P4)* |
| Meat and dairy substitutes omnivorous | Does the participant use meat and dairy substitutes for their child (≥4) on an OMD | *“We also don't buy that much meat, really those meat replacers that are a bit more expensive. And sometimes there is something on offer and then: oh nice, I'll take that with me. Then we'll try that.” (P4)* |
| Ready-made products vegan | Does the participant use ready-made (baby) products for their child (≥4) on a VD | *“Yes, those jars [ready-made infant food, that is indeed one of those things, that I think several mothers suffer from, the first one has not had them and the second has had them very often haha.” (P7)* |
| On the go, outdoors vegan | Does the participant use ready-made (baby) products for their child (≥4) on a VD when on the go or outside the home environment | *“Yes, with that very small one [child], I did indeed sometimes take a jar [ready-made infant food] with me.” (P7)* |
| Meat and dairy substitutes vegan | Does the participant use meat and dairy substitutes for their child (≥4) on a VD | *“Meat, yes I have to say that I do try to use unprocessed meat substitutes as much as possible, so just tofu and tempeh actually.” (P7)* |
| Ready-made products vegetarian | Does the participant use ready-made (baby) products for their child (≥4) on a VEGD | *“A nephew and niece from the village… at one point we saw them sitting with such an Olvarit jar [ready-made infant food] where the orange was behind the ears, so chemical. And then I looked at the ingredients list, then I said: we are not doing this. I've never bought an Olvarit jar or anything like that.” (P13)* |
| On the go, outdoors vegetarian | Does the participant use ready-made (baby) products for their child (≥4) on a VEGD when on the go or outside the home environment | *“Apple wedges, I do also carry along and… those stupid squeeze fruit things and of course, they are great.” (P13)* |
| Meat and dairy substitutes vegetarian | Does the participant use meat and dairy substitutes for their child (≥4) on a VEGD | *“Yes, they like that. Fake fish fingers or fake schnitzel, yes, they really like that, for sure.” (P13)* |
| Fussy or easy eater omnivorous | Is the child (≥4) on an OMD a fussy or easy eater | *“My eldest is very easy and eats almost everything.” (P5)* |
| Fussy or easy eater vegan | Is the child (≥4) on a VD a fussy or easy eater | *“Especially the oldest child, he really doesn't eat that much at dinner… I think that will sort itself out in the end.” (P22)* |
| Fussy or easy eater vegetarian | Is the child (≥4) on a VEGD a fussy or easy eater | *“They really eat everything: from my in-laws' Indonesian food, to potatoes, pastas.” (P13)* |
| Moment of introducing first bites omnivorous | The moment when parents introduced the first bites to their child (≥4) on an OMD | *“I think with half a year we started with some fruit snacks now and then and uhm, slowly expanding with some bread and stuff.” (P16)* |
| Moment of introducing first bites vegan | The moment when parents introduced the first bites to their child (≥4) on a VD | *“My eldest child was ready earlier, so he actually got a bite at four months.” (P24)* |
| Moment of introducing first bites vegetarian | The moment when parents introduced the first bites to their child (≥4) on a VEGD | *“With the eldest, I think we started with 4 months, now and then with something funny, because she found everything exciting and fun and at 6 months so real, just a real bite a day.” (P13)* |
| **Supplements** | Supplements that are used by either the participant or their child(ren) | |
| Own supplements | Does the participant use supplements when not pregnant and which supplements | *“B12 and vitamin D.” (P27)* |
| Own supplements during breastfeeding | Does/did the participant use supplements during breastfeeding and where these different from their standard supplements | *“When she was still exclusively breastfed, I took the Solgar's [brand] prenatal vitamins a little longer because… it is also specifically stated for the pregnancy and the lactation period. And from the moment she ate more solid food than that she drank milk, I stopped that and just started taking my normal multivitamins again.” (P3)* |
| Supplements child vegan | Does the participant's child(ren) on a VD use supplements and which supplements | *“They get B12 and vitamin D and omega 3.” (P24)* |
| Supplements child vegetarian | Does the participant's child(ren) on a VEGD use supplements and which supplements | *“That is vitamin d according to me, from Davitamon [brand] we now have.” (P13)* |
| Supplements child omnivorous | Does the participant's child(ren) on an OMD use supplements and which supplements | *“Well, they only get vitamin d and that is the standard of course, but they don't eat vegan either.” (P4)* |
| **Attitude** | | |
| Extra health check in general | How does the participant perceive doing extra health checks due to the VD and has she done them | *“I have my blood checked once a year, just to make sure everything is fine.” (P3)* |
| Healthiness diet in general | How does the participant perceive the healthiness of a VD | *“I just really think it is healthier to just not eat all that, yes antibiotics-injected meat and that fish that is now full of mercury.” (P11)* |
| Cons diet in general | Does the participant experience cons of a VD | *“I think mainly just people's opinions. And that you always just have to be aware of it and explain it... you will get questions anyway and people who don't like it [VD].” (P26)* |
| Completeness diet | How does the participant perceive the completeness of a VD | *“About completeness, yes 100%. I also find it special that there is indeed an idea that it could not be fully-fledged, because well, what I just said is: yes, I am the best example myself.” (P7)* |
| Pros diet in general | Does the participant experience pros of a VD | *“We [participant and partner following VD] are both quite strong in the sense that we are not sick very often… I used to get sick once or twice a year and I haven't had that for a number of years now.” (P20)* |
| Perspective on an OMD | What is the participant's perspective on an OMD | *“I think: it's so illogical that we live so much yes, just for our own pleasure, because it's not necessary [to eat animal-based products], it's really just a taste thing, as far as I'm concerned.” (P7)* |
| **Attitude children** | | |
| **Attitude diet choice child omnivore** | Participant's attitude about diet choice when child follows an OMD | |
| Extra health checks child | Participant's attitude about doing extra health checks for the child because of the VD when the child follows an OMD | *“But of course, children grow very fast, so I would… find it very exciting, even though I know a lot about whether you can indeed do it [following a VD] well enough, so that they do not get shortages of one or the other. So, if yes, if we were indeed 100% plant-based, then I would at least want to keep a close eye on that and I don't think with blood tests in the first instance, but by perhaps going to the health center once more to weigh and to measure it.” (P5)* |
| Healthiness diet | Participant's perception of the healthiness of an OMD for a child | *“I also believe that dairy and eggs and stuff, would actually also have quite a lot of adverse health effects” (P5)* |
| Healthiness vegan breastfeeding | Participant's perception of the healthiness of breastfeeding of mothers following a VD | *No citation* |
| Healthiness VD for child | Participant's perception of the healthiness of a VD for a child when the child follows an OMD | *"I think that [following a VD] is fine for them. I don't know if it's necessarily better for them, but I don't think it's necessarily worse. Otherwise, I wouldn't have to do it myself… With children it's a bit: they grow from the carbohydrates, so as soon as they get the carbohydrates, it really doesn't matter if they also eat that piece of meat or those 3 pieces of broccoli." (P4)* |
| Cons of an OMD for child | Participant's perception of the cons of an OMD for a child | *No citation* |
| Cons of a VD for child | Participant's perception of the cons of a VD for a child when the child follows an OMD | *“I think it might be difficult that she is different from others… that she experiences it that way.” (P19)* |
| Completeness diet | Participant's perception of the completeness of a VD for a child when the child follows an OMD | *“For my youngest, for example, I would say:… during primary school age, then rather vegetarian than completely plant-based. Just to build in a little bit of… security for myself. Just a little guarantee that they really do get everything they need.” (P5)* |
| Pros of an OMD for child | Participant's perception of the pros of an OMD for a child | *“I really like that they can taste everything and we are very free with everything, including sugar for example. Yes, you can, it's all in moderation, but you can really try it all once and if it's a party or we're at grandpa and grandma's or eating out… then we don't make a fuss about it.” (P4)* |
| Pros of a VD for child | Participant's perception of the pros of a VD for a child when the child follows an OMD | *No citation* |
| **Attitude diet choice child vegan** | Participant's attitude about diet choice when child follows a VD | |
| Extra health checks child | Participant's attitude about doing extra health checks for the child because of the VD | *"I do think that at some point... I don't really know at what age it is normal to have blood samples taken. But yes, at some point, I would like to have her tested once in a while… Just to be a little sure." (P26)* |
| Healthiness VD for child | Participant's perception of the healthiness of a VD for a child | *“I am convinced that a vegan and preferably whole food, plant-based [diet]… is simply the healthiest… I also want to pass that on to my son.” (P6)* |
| Healthiness vegan breastfeeding | Participant's perception of the healthiness of breastfeeding of mothers following a VD | *“I did have that feeling: oh, you see my breastfeeding is super, super good and that's probably because, because I am vegan… Well, that's probably because I just eat completely plant-based, but of course you don't know that. That is more of an assumption from yourself that you think: oh, that might play a part.” (P11)* |
| Cons of a VD for child | Participant's perception of the cons of a VD for a child | *“I think it might be difficult that she is different from others… that she experiences it that way.” (P19)* |
| Completeness diet | Participant's perception of the completeness of a VD for a child | *“I also think that… certainly in the phase in which they grow like this, there are also just good nutrients in a chicken egg, so it is not that it [animal-based products] is necessarily very bad for their health or that I have the idea that if she gets dairy once in a while, she will get something out of it… It's more that I don't support it ethically and that I believe and also did on the basis of research, that there are full-fledged [vegan] alternatives. So that is more a matter of feeling and also based on the conversation I had with the dietitian.” (P3)* |
| Pros of a VD for child | Participant's perception of the pros of a VD for a child | *“I think it is an advantage that you ingest much less bio accumulative toxic substances, because… the world is quite toxic these days. It also accumulates in animals… If you eat those animals, you get that in higher concentrations than if you only eat the plant.” (P10)* |
| **Attitude diet choice child vegetarian** | Participant's attitude about diet choice when child follows a VEGD | |
| Extra health checks child | Participant's attitude about doing extra health checks for the child because of the VD/VEGD | *“I honestly haven't thought of that [doing blood checks] at all for the kids... I find it funny that I never thought about that. I also honestly think that it is not necessary, because I think they are getting their nutrients… as long as I don't get signs of tiredness or pale…If there's something wrong with your child, then of course you'll go after it anyway… I see them all doing very well, so then I think: no, I'm not just going to have a blood test or something like that.” (P2)* |
| Healthiness diet | Participant's perception of the healthiness of a VEGD for a child | *“I think that we eat somewhat healthier. Well at least we eat healthier than the average Dutchman… I think we eat a bit more vegetables. Much more legumes than the average Dutch person, which is of course also very healthy.” (P1)* |
| Healthiness VD for child | Participant's perception of the healthiness of a VD for a child | *“I just don't think it's necessary at all for him to eat something that animals have suffered for and I also just really, really don't think he needs it, because there are so many [vegan] alternatives.” (P25)* |
| Healthiness vegan breastfeeding | Participant's perception of the healthiness of breastfeeding of mothers following a VD | *“If I have understood correctly… it would rather be first, the breastfeeding must be good. After that it's your turn as a mother, so it may be that I run into shortages, for example, but I didn't worry so much about the milk… That comes first, that's where all the nutrients that are needed for my child go. I've also read somewhere that yes, if you breastfeed for a long time and a VD, that really doesn't go together and that hit me a bit, that scared me a bit. And then I did some further research and came to the conclusion: I think it can be done really well. Yes, you just have to take good care of yourself… I really don't think that my child is short of something… As soon as I fall short of something, I will of course notice.” (P1)* |
| Cons of a VEGD for child | Participant's perception of the cons of a VEGD for a child | *No citation* |
| Cons of a VD for child | Participant's perception of the cons of a VD for a child when the child follows a VEGD | *“I think that if you really want your children to eat 100% plant-based, then that is difficult, because that is not always possible and then well, then I think you will also be looked at strangely.” (P1)* |
| Completeness diet | Participant's perception of the completeness of a VD for a child when the child follows a VEGD | *No citation* |
| Pros of a VEGD for child | Participant's perception of the pros of a VEGD for a child | *“I really see that, of course they are stubborn, they want to eat some things better than other things, but they eat really well in relation to the peers… I really like to see that. I mean, of course, every parent wants the child to eat well.” (P13)* |
| Pros of a VD for child | Participant's perception of the pros of a VD for a child when the child follows a VEGD | *“I also think that yes, that it [eating mostly plant-based] can have benefits… the climate of course, he has to live here for a very long time. Why should I start giving him something that slowly destroys the world? Yeah, that's a bit counterintuitive to me too.” (P25)* |
| **Subjective norm** | | |
| **Subjective norm vegan in general** | What is the participant's perception of how relevant other value that the participant follows a VD and motivation to comply | |
| Ask advice on VD | Did the participant ask advice on their VD and to whom | *"I think it was around April or May that I initially had contact with the dietitian and that was actually for two reasons: and because I wanted to eat completely plant-based… the other side of that was that with my other two children after giving birth, I also lost a lot of weight… and I just didn't feel comfortable with that.” (P2)* |
| Influence of opinions on diet choice | Did the opinions from relevant others about following a VD influence the participant's diet choice | *“I: Have these opinions influenced your [VD] choices or have you been able to refute them every time?”*  *“P: No, it's just how I do it and I just still… fully support the ethical importance [of a VD].” (P2)* |
| Opinions surrounding | Did the participants experience opinions from relevant others on following a VD | *“I have a colleague at work who is also vegan… and at that clinic they are somewhat okay. But at the other clinic… I'm the only one [following a VD], everyone eats meat, no vegetarians. They always find that [VD] a bit strange and difficult.” (P16)* |
| Opinions health professionals | Did the participant experience opinions from health professionals about following a VD | *“And my GP in X [city], who also knows that we eat plant-based and they also know that I get my blood tested once a year, just to make sure everything is okay.” (P3)* |
| What do you think of these opinions | What does the participant think of these opinions or what is the participant's reaction to these opinions | *“I also notice that I care less and less, because it [VD] is just something I stand for and I just want to live what I think is important.” (P12)* |
| **Subjective norm children** | | |
| **Subjective norm child omnivorous** | What is the participant's perception of how relevant other value adopting an OMD for their child(ren) and motivation to comply | |
| Opinions surrounding about non-vegan raising | Did the participants experience opinions from relevant others on not raising a child on a VD | *“Yes, they all thought that [raising child on an OMD] was a logical decision haha.” (P4)* |
| Influence of opinions on diet choice child | Did the opinions from relevant others about raising a child on a VD influence the participant's diet choice of the child | *No citation* |
| Opinions health professionals | Did the participant experience opinions from health professionals about raising a child on a VD | *No citation* |
| What do you think of these opinions | What does the participant think of these opinions or what is the participant's reaction to these opinions | *No citation* |
| Allowing products of animal origin | Does the participant allow the child to eat products of animal origin when the child follows an OMD | |
| At others, out of the home environment | Does the participant allow the child to eat products of animal origin when eating at others, outside the home environment, when the child follows an OMD | *“I'd rather, they aren’t restricted [in their diet] now. That it's not like they're going to children's parties and saying: oh, mommy won't let me eat that [non-vegan food] or something like that, you know.” (P4)* |
| Making own choices | What does the participant state about letting the child make own choices about her/his diet when the child follows an OMD | *“I think anyway, I think it's important that in the end, it's their own choice.” (P5)* |
| Explaining the child what products of animal origin are/consist of | Does the participant explain to their child(ren) what products of animal origin are/consist of when the child follows an OMD | *“For example, if they ask questions about why I don't eat that [animal-based product]… in the end, I do want to say it to them: look, this is how the world works now. And hopefully the world will have improved a bit by then haha. But I certainly intend to [inform them].” (P4)* |
| Child asks it | How did the participant respond when the child asked about wanting to consume products of animal origin when the child follows an OMD | *“Occasionally they eat meat, but I try to limit it and when I do the shopping, very rarely that I buy meat and that is really for special days or something or if you know when someone has a birthday and they ask specifically for something then they get that.” (P5)* |
| Intention child asks it | How does the participant intent to respond when the child asks about wanting to consume products of animal origin when the child follows an OMD | *No citation* |
| **Subjective norm child vegan** | What is the participant's perception of how relevant other value adopting a VD for their child(ren) and motivation to comply | |
| Ask for advice on child’s diet choice | Did the participant ask advice on the diet choice of their child and to whom | *"When my daughter was six months old and started with solid food, I scheduled 1 consultation with a plant-based dietitian and that's more because children can eat quite irregularly. My daughter too… she eats, one day she eats 4 slices of bread and the next day only 1 and that sometimes gives you a bit of a restless feeling because you want her to eat everything.” (P3)* |
| Health center and reaction | Did the participant tell the health center that the child is raised on a VD and what was their reaction | *“No, no. I didn't [tell the health center that the child is raised on a VD]… I didn't feel like being judged in advance… But if they really asked specifically, I would say so. But it was not asked.” (P23)* |
| Childcare | Did the participant tell the childcare that the child is raised on a VD and their reaction | *“They were very easy about it, because it is quite an anthroposophical childcare where he goes… and everything is organic. I think vegetarian too. Not vegan, but vegetarian and they always bake their own bread there, and that, so they are quite wholesome in my opinion.” (P17)* |
| Opinions surroundings about vegan raising | Did the participants experience opinions from relevant others on raising a child on a VD | *“I can remember a colleague who said when I was pregnant: well, I would occasionally give meat. Kind of like: is really sad [to raise child on a VD]. Very occasionally we have to deal with it, but actually very, very little.” (P11)* |
| Influence of opinions on diet choice child | Did the opinions from relevant others about raising a child on a VD influence the participant's diet choice of the child | *“No. I did take a closer look to see if there was anything to read online about the health effects of the [vegan] diet in small children and pregnancy.” (P17)* |
| Opinions health professionals | Did the participant experience opinions from health professionals about raising a child on a VD | *“He also happened to be in the hospital recently for some tests and then I do indicate that he eats plant-based, but there is really no response. I think it's just becoming more and more normal.” (P15)* |
| What do you think of these opinions | What does the participant think of these opinions or what is the participant's reaction to these opinions | *“When I just gave birth I found it quite… tiring always having to defend myself. That's just not fun.” (P3)* |
| Allowing products of animal origin | Does the participant allow the child to eat products of animal origin when the child follows a VD | |
| At others, out of the home environment | Does the participant allow the child to eat products of animal origin when eating at others, outside the home environment, when the child follows a VD | *“We notice that [asking to prepare] vegan food, then you just ask quite a bit of people of course. So, for example at childcare or for friends, we say: vegetarian.” (P7)* |
| Making own choices | What does the participant state about letting the child make own choices about her/his diet when the child follows a VD | *“In principle, we just have to see how it [raising child on a VD] goes, of course, but what we want to say in any case is that we eat plant-based at home and that we have these reasons. In particular, the ethical side of it and yes, what she ultimately does with it, she must of course just decide for herself.” (P18)* |
| Explaining the child what products of animal origin are/consist of | Does the participant explain to their child(ren) what products of animal origin are/consist of when the child follows a VD | *“I am such that I am not forbidding my children, so if my son or daughter later says: I still want to eat meat or milk, then he or she may do so, but I will provide him with the correct information. So, at the age that he can make that choice, he can't do that yet because he's 2.5.” (P2)* |
| Child asks it | How did the participant respond when the child asked about wanting to consume products of animal origin when the child follows a VD | *“If my son asks somewhere if he can have a slice of cow cheese, then I allow him to have a slice of cow cheese, we just never have it at home.” (P9)* |
| Intention child asks it | How does the participant intent to respond when the child asks about wanting to consume products of animal origin when the child follows a VD | *“Yes, then that [consuming a product of animal origin] is fine. I also think that it will come, because you also want to try everything, of course.” (P12)* |
| **Subjective norm child vegetarian** | What is the participant's perception of how relevant other value adopting a VEGD for their child(ren) and motivation to comply | |
| Health center and reaction | Did the participant tell the health center that the child is raised on a VEGD and what was their reaction | *“Yes, they [health center] know. They have written it down. It has been noted.” (P13)* |
| Childcare | Did the participant tell the childcare that the child is raised on a VEGD and their reaction | *“Yes, I registered him [at childcare], yes. But no contact yet.” (P28)* |
| Opinions surrounding about non-vegan raising | Did the participants experience opinions from relevant others on not raising a child on a VD | *“Yes that, well yes, it makes a difference there too I think that the children eat vegetarian, because I think there are quite a few people who have an opinion about children eating completely vegan. In our area, actually, we also know a lot of vegetarians, so it's really quite normal.” (P1)* |
| Influence of opinions on diet choice child | Did the opinions from relevant others about raising a child on a VD influence the participant's diet choice of the child | *No citation* |
| Opinions health professionals | Did the participant experience opinions from health professionals about raising a child on a VD | *No citation* |
| What do you think of these opinions | What does the participant think of these opinions or what is the participant's reaction to these opinions | *“We just don't get any comments from anyone, so that's very nice anyway.” (P1)* |
| Allowing products of animal origin | Does the participant allow the child to eat products of animal origin when the child follows a VEGD | |
| At others, out of the home environment | Does the participant allow the child to eat products of animal origin when eating at others, outside the home environment, when the child follows a VEGD | *“Suppose he goes to a children's party. And yes, for example… they want a hamburger, yes, then I will not say that he is not allowed. He can eat that.” (P25)* |
| Making own choices | What does the participant state about letting the child make own choices about her/his diet when the child follows a VEGD | *“I don't want to dictate that she doesn't eat meat anymore, but I also don't want to dictate what she can eat. So, I really want to leave it to her.” (P27)* |
| Explaining the child what products of animal origin are/consist of | Does the participant explain to their child(ren) what products of animal origin are/consist of when the child follows a VEGD | *“I think in a year or so, then maybe I can tell a few more details. And yes, that also goes step by step, because than you don't want to tell very miserable stories right away… you also notice it with the children. At some point they will ask certain questions. And yes, if they ask questions, they will get honest answers.” (P1)* |
| Child asks it | How did the participant respond when the child asked about wanting to consume products of animal origin when the child follows a VEGD | *No citation* |
| Intention child asks it | How does the participant intent to respond when the child asks about wanting to consume products of animal origin when the child follows a VEGD | *“At some point they will start making choices themselves…*  *If they are with peer friends and the sausages are not vegetarian, but normal and they say: I want that. Yes, we have yet to see when that age is.” (P13)* |
| **Perceived behaviour control** | | |
| Succeeding in sticking to this diet | Does the participant think that she can succeed in sticking to a VD | *“I don't find it difficult to keep up the eating pattern… because I really feel this is the right decision. I find it more difficult that everyone thinks it's normal or something, to eat so many animals and that everyone doesn't think about the consequences of their behavior.” (P10)* |
| **Perceived behaviour control factors** | The factors that make it easier or more difficult to follow a VD | |
| Availability, stores | The availability of vegan food products in stores | *“It is easier that there is now much more available and that it is very well indicated on products.” (P22)* |
| Economic | The economic aspects of a VD | *“Yes, that [costs of vegan products] in itself, is fine. We also have… just a double income, so we haven't had too many issues with that. I can imagine that this is difficult for others.” (P14)* |
| Family, partner | The family and partner of the participant | *“I also have it very easy in that respect, because of course my boyfriend also eats vegan.” (P15)* |
| Knowledge | The knowledge of the participant or others about a VD | *“I think it just has to do with knowledge sharing, so to speak. The information more accessible and really in a bite-sized way, because I, I then have a scientific background so I, I can still read a scientific article but well… that is sometimes quite difficult.” (P6)* |
| Enjoyment of cooking | The enjoyment of cooking | *“I'm not really a kitchen princess, but I have a number of very good vegan cookbooks… from which I can get very tasty recipes, so that also makes it a lot easier.” (P20)* |
| Media | The media | *“I do think that the media has contributed to opening up the conversation [about plant-based diets], and that people, the flexitarians, have no meat for a day, that's all right now.” (P13)* |
| Opinions | Opinions from others in the surrounding of the participant | *“In the social field, it can be complicated. And you have to… want to engage in it [discussions about a VD]… what I also found out is, of course, you don't always have to explain it, so I can just say: oh no, I'm not allowed to do that [eating non-vegan products].” (P5)* |
| Politics, lobby | Politics and lobby about food | *“I think that's one of the main things that makes the transition to vegan difficult, is just the lobby. It is not for nothing that now the oat milk with VAT will become super expensive and then chocolate milk, which contains sugar, will not receive a sugar tax. Yes lobby.” (P2)* |
| City, village, country | Living in a specific city, (rural) village or country | *“We no longer go on holiday to France, because it is very difficult there.” (P20)* |
| Eating out | Eating out when following a VD | *“Suppose you're going out for dinner with colleagues or something. You can always say: well, I'm plant-based. They usually have something… not always much choice, but that doesn't matter at all.” (P18)* |
| Friends | Friends of the participant | *“Friends of ours, are very willing to dig into that [preparing vegan food] when they cook for us, so that's yeah, that's really nice.” (P11)* |
| Work | The work environment of the participant | *“I actually only find it difficult in those, yes kind of cliché situations, that you have a course day and then I indicate it [following a VD]… but very often… you still get a cheese sandwich.” (P7)* |
| **Perceived behaviour control children** | | |
| Succeeding in sticking to this diet vegan child | Does the participant think that she can succeed in sticking to a VD for a child | *“Honestly, I don't know. I do think that we can just offer her full-fledged [vegan] food. I don't have any doubts about that. I think it also depends a bit on how she develops in terms of character, will she be very opposed to it [VD]? Or… is she also supporting it? And especially when she's a bit older that you just have to be able to have a conversation about that and also respect her choice… If at some point she is 15 and she really wants to fry a piece of meat, maybe we should just be okay with that too.” (P18)* |
| **Perceived behaviour control factors children** | | |
| PBC child omnivorous | The factors that the participant thinks that make it easier or more difficult to raise a child on a VD while the participant's child(ren) is/are raised on an OMD | *“Sometimes, I get a [vegan] chicken burger from Beyond Meat or something and those things are really expensive, but then you know, I have [buy] it for myself [only]. But if we with the five of us… when the children are a bit bigger and suppose we all switch over together [to a VD], then I think that will really be a luxury for an occasional instead of once a week or something.” (P5)* |
| PBC child vegan | The factors that the participant thinks/experiences that make it easier or more difficult to raise a child on a VD while the participant's child(ren) is/are raised on a VD | *“Yes, and that is quite difficult sometimes, because then he gets a sandwich with a vegan sausage and a glass of soy milk. And then the child sitting next to him, he gets a sandwich with a different [animal-based] sausage or cheese or something. And then he can't taste a bite, so to speak.” (P17)* |
| PBC child vegetarian | The factors that the participant thinks that make it easier or more difficult to raise a child on a VD while the participant's child(ren) is/are raised on a VEGD | *“It makes a difference there too I think that the children eat vegetarian, because I think there are quite a few people who think something about children eating completely vegan.” (P1)* |
| **Knowledge** | | |
| Gain knowledge vegan in general | What resources did the participant consult for gaining knowledge on a VD in general | *“I have done so much research into it [VD] myself, read so much about it.” (P23)* |
| Gain knowledge for child | What resources did the participant consult for gaining knowledge on a VD for a child | *“Of course, I had in advance… a book, I think it was an American book, about how to deal with raising children vegan… It also said that… dietitians association say that if you just eat well balanced, that a vegan lifestyle is suitable for all age groups.” (P14)* |
| Knowledge health professionals | Any statements or experiences about health professionals' knowledge of a VD | *‘I think there is still a lot to be gained in healthcare and when it comes to the knowledge of professionals [about a VD] and not everyone has to master it completely, but that you [healthcare professionals] know: okay, I at least know where to refer to for the correct information.” (P18)* |
| Sufficient knowledge in general | Does the participant think that she has sufficient knowledge about a VD in general | *“I: And did you then have the idea that you thought: now I really have gained enough knowledge to be able to handle this [VD] properly?”*  *“P: Yes, yes sure. So, I've never actually been unconfident in being vegan.” (P2)* |
| Sufficient knowledge for child | Does the participant think that she has sufficient knowledge about a VD for a child | *“I: Do you think that all in all you really have enough knowledge to raise your children in this way [on a VD]?”*  *“P: Yes, in any case, to make responsible choices yes.” (P1)* |

P, participant; OMD, omnivorous diet; VD, vegan diet; VEGD, vegetarian diet
